# Oral Vaccination Approaches for Anti-SHIV Immunity

**DOI:** 10.3389/fimmu.2021.702705

**Published:** 2021-06-21

**Authors:** Erandi Velarde de la Cruz, Lingyun Wang, Deepanwita Bose, Sailaja Gangadhara, Robert L. Wilson, Rama R. Amara, Pamela A. Kozlowski, Anna Aldovini

**Affiliations:** ^1^ Department of Medicine, Boston Children’s Hospital, Boston, MA, United States; ^2^ Department of Pediatrics, Harvard Medical School, Boston, MA, United States; ^3^ Emory Vaccine Center, Yerkes National Primate Research Center, Emory University, Atlanta, GA, United States; ^4^ Department of Microbiology and Immunology, Emory School of Medicine, Emory University, Atlanta, GA, United States; ^5^ Department of Microbiology, Immunology and Parasitology, Louisiana State University Health Sciences Center, New Orleans, LA, United States

**Keywords:** HIV, oral vaccine, poliovirus vector, mucosal immunity, AIDS, SHIV vaccine

## Abstract

We modified a Sabin Oral Poliovirus Vaccine (OPV) vector to permit secretion of the antigens of interest with the goal of improving anti-HIV Env humoral responses in a SHIV mucosal immunization composed of DNA and recombinant OPVs. We evaluated stimulation of systemic and mucosal cell-mediated and humoral immunity in Rhesus macaques by two regimens, both involving a prime with a SHIV_BG505 _DNA construct producing non-infectious particles formulated in lipid nanoparticles, administered in the oral cavity, and two different viral vector boostings, administered in the oral cavity and intestinally.** **Group 1 was boosted with rMVA-SHIVBG505, expressing SIV Gag/Pol and HIV_BG505_ Env. Group 2 was boosted with a SHIV_BG505_-OPV vaccine including a non-secreting SIV_mac239_CA-p6-OPV, expressing Gag CA, NC and p6 proteins, and a HIV_BG505_C1-V2-OPV, secreting the C1-V2 fragment of HIV Env_BG505_, recognized by the broadly neutralizing antibody PG16. A time course analysis of anti-SHIV Gag and Env CD4+ and CD8+ T-cell responses in PBMC and in lymph node, rectal, and vaginal MNC was carried out. Both regimens stimulated significant cell-mediated responses in all compartments, with SHIV_BG505_-OPV immunization stimulating more significant levels of responses than rMVA- SHIV_BG505_. Boolean analysis of these responses revealed predominantly monofunctional responses with multifunctional responses also present in all tissues. Stimulation of antibody responses was disappointing in both groups with negative anti-SHIV IgG in plasma, and IgA in salivary, rectal and vaginal secretions being restricted to a few animals. After repeated rectal challenge with SHIV_BG505_, two Group 1 animals remained uninfected at challenge termination. No significant differences were observed in post-infection viral loads between groups. After the acute phase decline, CD4+ T cell percentages returned to normal levels in vaccinated as well as control animals. However, when compared to controls, vaccinate groups had more significant preservation of PBMC and rectal MNC Th17/Treg ratios, considered the strongest surrogate marker of progression to AIDS. We conclude that the vaccine platforms used in this study are insufficient to stimulate significant humoral immunity at the tested doses and schedule but sufficient to stimulate significant mucosal and systemic cell-mediated immunity, impacting the preservation of key Th17 CD4+ T cells in blood and rectal mucosa.

## Introduction

Vaccine development for prevention of AIDS has been attempted since the discovery of HIV-1, exploring numerous available platforms, yet clinical trials carried out so far have been disappointing. The RV144 trial, evaluating the efficacy of four doses of inactivated HIV-recombinant ALVAC pox virus plus two of recombinant monomeric HIV gp120, showed that the vaccine had 30% efficacy in preventing infection, with protection being linked to antibodies against the V1V2 region of gp120 (1–4). Protection was limited and declined over time, due to the declining antibody (Ab) response (5). This vaccine had low levels of T-cell immunogenicity and vaccination did not control viremia or loss of CD4+ T cells in the vaccinees that contracted HIV (2, 6–8). However, polyfunctional CD4+ and CD8+ T-cell responses correlated with decreased HIV risk and, at the same time, a sieve effect on transmitted viruses induced by the cellular responses was noted (5, 9, 10). Recently results from additional boosting of participants in the RV144 trial have become available. Boosting increased the antibody responses and affinity maturation to levels higher than those in the original trial but these responses were not long-lasting and did not further increase with subsequent boosts (11–14). Mucosal IgG levels against different Env immunogens correlated with the plasma IgG levels (11). In the trial RV305, the vaccine boost expanded anti-HIV env CD4 binding site antibodies capable of neutralizing tier 2 primary isolates (15). A caveat in the interpretation of the protection data was also raised and highlighted the role of trial participants engaged in low- *vs.* high-risk behavior in affecting the outcome of vaccine efficacy (16). Importantly, the ALVAC–gp120 regimen was very recently shown not to prevent HIV-1 infection in vaccinated individuals hat were part of a trial in South Africa, although in this trail the adjuvant used in conjunction with gp120 was different than that used in RV144 (17).

Macaque immunization experiments that failed to provide sterilizing immunity showed that some infection protection or delayed onset of disease can be observed if anti-SIV cell-mediated immunity is present at the time of exposure and infection [(18–24) and references therein]. Although initial clinical trials based on vaccines that stimulated T-cell immunity were disappointing, the utility of the stimulation of this arm of the immune system in conjunction with induction of antibodies with diverse functions is being reconsidered in light of data from elite controllers and because there are advantages provided by cell-mediated responses to HIV, including the ability to target broad, mutationally constrained epitopes of multiple HIV proteins without a requirement for somatic hypermutation, which may allow for more standard prime–boost vaccine regimens (25). Mucosal responses may also protect against cell-associated HIV transmission and be able to clear an infection before reservoirs are established. If antigen-specific immunological memory is not sufficient to prevent the establishment of chronic HIV-1 infection, a more successful approach could be based on vaccines that maintain high levels of effector memory cells that mimic the response induced by attenuated SIV viruses, while avoiding their drawback of persistent infection and disease. A recombinant CMV approach has provided this type of immunity that resulted in aborted infection, indicating that a persistent effector T-cell response can prevent the establishment of reservoirs (22, 26–28).

Data from preclinical vaccine trials support the notion that anti-Env broadly neutralizing antibodies (bNAbs) could be a critical component in addition to anti-HIV cell-mediated immunity to achieve large scale protection from persistent infection, as passive administration of neutralizing antibodies protects macaques from SHIV challenge (29). When high titer NAbs were induced by vaccination, protection from homologous rectal challenge was initially obtained but was lost as NAb titer declined (30). Anti-V1V2 Abs mediating ADCC have been identified as correlate of protection in the RV144 trial and there is now significant focus on this HIV Env domain as a critical vaccine component (1, 3, 6, 31–33). Cloning bNAbs from HIV-infected individuals indicates that significant Ab affinity maturation is necessary to move from poorly NAbs, encoded by germ-line Ab sequences, to higher affinity antibodies, capable of providing broad neutralization (34–37). Stimulation of bNAbs *via* vaccination has been a frustrating endeavor in the HIV vaccine field. The Env protein needs to fold into the appropriate trimeric conformation to stimulate NAbs and the Env gp140 SOSIP appears to induce tier 2 NAbs, capable of neutralizing tier 2 SHIV that are more representative of circulating HIVs (30, 38–42). Env immunogens capable of stimulating bNAbs are therefore required to have simultaneously ideal domains for appropriate structural conformation and the ability to drive the affinity maturation process towards the selection of bNAbs. Furthermore, a platform capable of inducing long-lasting titers of humoral immunity is also necessary. The ideal candidate antigens and vectors necessary to simultaneously achieve all these goals have not yet been identified and are the focus of many investigations. Furthermore, the mechanisms of protection in the RV144 trial are not fully understood and are possibly mediated by antibody-dependent-cell-mediated cytotoxicity (ADCC) and other non-NAb effector functions. The correlation with protection of these non-NAbs raises questions whether bNAbs should be the only relevant goal of a successful vaccine (14, 43).

It is possible that significant immunity at the site of HIV entry might permit control of local infection before it becomes systemic and therefore reduces the plasma Ab titer necessary for protection. Immunization at one mucosal site can lead to an immune response at other mucosal effector sites, as immunologically competent cells with homing receptors specific for mucosal sites circulate among different sites, but there are differences in the magnitude observed at different sites with maximal responses at the site of antigen exposure and present to a lesser degree at other mucosal sites, supporting the notion of compartmentalization of the mucosal immune system (44–50).

The exploration of the oral route of immunization is important for two reasons, one practical, the second due to its potential to provide a more tailored approach to HIV immunity and protection. Its practicality, when compared to systemic or other mucosal routes, resides in its simplicity of administration and therefore in its feasibility in settings with less-than-optimal health care resources. As for stimulation of a broad and diverse immunity against HIV that is present also at the sites of viral exposure, mucosal routes of immunizations are known to be better than systemic routes to achieve both mucosal and systemic immunity, with oral immunization being ideal for achieving immunity in the oral cavity and in the gastrointestinal tract (51). OPV has been the most successful and safe oral vaccine used in the world, critical to the goal of getting close to eradicating poliomyelitis (52). As it replicates in the intestinal tract and also reaches the systemic compartment, it is capable of inducing excellent responses at both sites that last at high titers for decades, even in the absence of re-exposure and could be a valuable vector for induction of broad humoral immunity, with stimulation of both antigen specific IgG and IgA in the rectal mucosa that could be significant to control HIV at the site of entry, as passively administered anti-HIV Env IgA mAbs have been shown to be highly effective for preventing mucosal SHIV infection in RM (53). In children, neutralization titers have been shown to correlate with duration of shedding, with titers being higher when shedding could still be detected 28 days after immunization while being absent or lower when shedding was shorter (54). Some concerns were expressed about recombinant OPV capability of inducing significant cell mediated immunity (55), therefore its use in combination with a DNA priming appeared to us an interesting approach.

Here we combined a SHIV_BG505_ recombinant DNA prime with a boost composed of two recombinant OPVs, one expressing the SIV Gag CA-p6 fragment, containing the SIV capsid (CA) and nucleocapsid (NC) proteins, and one secreting a fragment of the HIV Env, covering the C1-V2 region and recognized by a bNAb, from the infected cell. This regimen was compared to one where the boosting vaccine was a recombinant SHIV_BG505_-MVA. We report the immune responses induced by these regimens in the Rhesus macaque animal model and their effect on a SHIV rectal challenge.

## Materials and Methods

### Construction of SHIV_BG505_ Vaccines

The plasmid pSHIV_BG505_Vacc3 used in the vaccination is a derivative of pVacc7 (56) in which a fragment containing the SIV_mac239_ *env* coding sequence was replaced by a corresponding fragment that includes the HIV_BG505_ *env* coding sequence, obtained by PCR amplification from a HIV-1 BG505 Env Expression Vector (BG505.W6M.ENV.C2, NIH-AIDS Reagent Program cat. No. ARP-11518) (57) with primers carrying SphI and NcoI restriction sites and cloned in a similarly digested pVacc7. The DNA sequence was confirmed by sequencing and the profile of the non-infectious viral particle produced by the construct was evaluated by cell lysate Western blot using a macaque SHIV-positive plasma. pSHIV_BG505_Vacc3 DNA was produced and purified at Aldevron Biotechnology (Fargo, ND). The DNA amount of pSHIV_BG505_Vacc3 used in one vaccine dose (1 mg) was formulated in 1 ml of 20 mM DOTAP (1,2-dioleoyl-3-trimethylammonium-propane, cholesterol (1:1) that forms cationic liposomes (formulation made by FormuMax Scientific Inc.Sunnyvale CA).

rMVA-SHIV-BG505 Immunogen (10^8^ pfu/dose/animal), expressing SIV_mac239_ Gag, Pol and HIV_BG505_ Env was developed using HIV_BG505_ Env sequence (Gen Bank accession: KU958484.1) with E64K-A316W-T332N-A433P, SOSIP mutations (A501C, T605C, I559P) and was codon optimized for MVA-mediated expression. This sequence was synthesized from GenScript and was cloned using Xmal site in pLW-73 with an independent mH5 promoter. It was subsequently recombined and developed as previously described (58) into MVA essential region expressing SIV Gag and Pol at Del III (provided by B.Moss) between genes I8R and G1L. Gag and Env expression in rMVA/SHIV-BG505 infected cells were confirmed by western blotting and flow cytometry. Viral stock for immunizations was purified from rMVA/SHIV infected DF1 cell lysates using 36% sucrose cushion.

**Figure 1:**
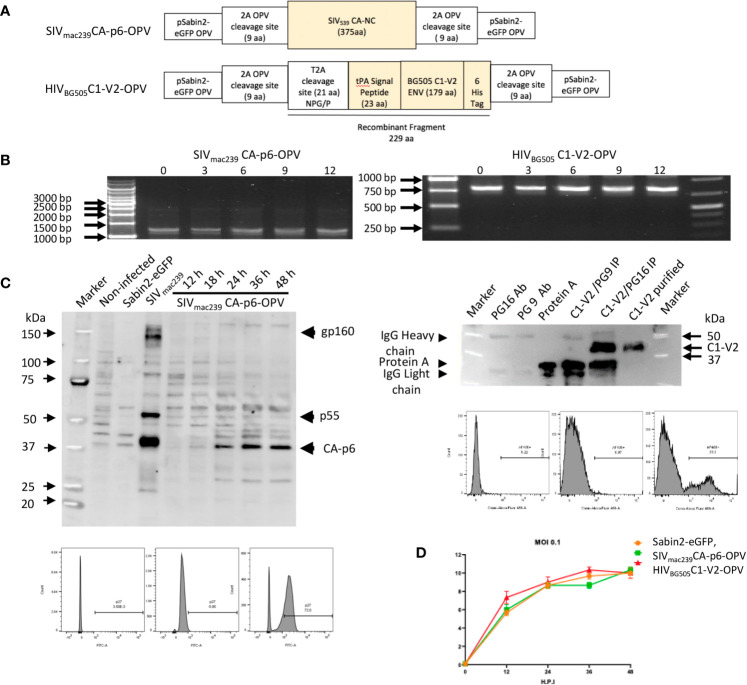
**(A)** Schematic diagram of Recombinant OPVs: orange boxes illustrate the SHIV recombinant antigens that are ultimately produced by OPV. The SIV_mac239_CA-p6 sequence was inserted between two 2A protease cleavage sites in pSabin2-eGFP, replacing *gfp* to generate SIV_mac239_CA-p6-OPV. The corresponding protein becomes expressed intracellularly once the cleavage of the OPV polyprotein occurs. The HIV_BG505_ Env C1-V2 region was cloned linked to tPA signal peptide that permits its secretion to generate HIV_BG505_C1-V2-OPV. The recombinant fragment amino acid sequence, starting and ending with polio protease cleavage sequences, with polio protease TTY**/**G and T2A NPG/P cleavage indicated by a bar, and with HIV Env sequence underlined, is the following: GLTTY**/**GFGHGGGGGGSRLEGSGEGRGSLLTCGDVEENPG**/**PMDAMKRGLCCVLLLCGAVFVSASAENLWVTVYYGVPVWKDAETTLFCASDAKAYETEKHNVWATHACVPTDPNPQEIHLENVTEEFNMWKNNMVEQMHTDIISLWDQSLKPCVKLTPLCVTLQCTNVTNNITDDMRGELKNCSFNMTTELRDKKQKVYSLFYRLDVVQINENQGNRSNNSNKEYRLINCNTSATQACPKVSFHHHHHHVDGLTTY/GFGH. **(B)** Stability of passaged recombinant OPVs: RT-PCR to detect recombinant OPV expression in RNA of infected cells after virus passage. Selective passages from 0 to 12 are reported. **(C)** Left top panel: Western blot of cell lysates from 293T cells: non-infected (lane 2), infected with Sabin2-eGFP (lane 3), transfected with SIV_mac239_ DNA (lane 4), infected with SIV_mac239_CA-p6-OPV (lanes 5-9, harvested at 12 to 48 hrs after infection). A SHIV-infected monkey serum was used as primary antibody. Left bottom panels: flow cytometric analysis of 293T infected cells stained with an anti-SIV p27 antibody (unstained, DAPI stained, DAPI+anti-p27 panels). Right top panel: Detection of the HIV_BG505_C1-V2 fragment by Western blot, probed with anti-HIS mAb: mAb PG16 (lane 2); mAb PG9 (lane 3), Protein A (lane 4), HIV_BG505_C1-V2 fragment, purified from tissue culture supernatant after HIV_BG505_C1-V2-OPV infection and immunoprecipitated with NAb PG9 (lane 5) or with NAb PG16. (lane 6), purified HIV_BG505_C1-V2 (lane 7). Right bottom panels: flow cytometric analysis of 293T infected cells stained with an anti-HIS mAb (unstained, DAPI alone staining, DAPI+anti-HIS staining panels). **(D)** Growth curve of recombinant OPVs in 293T cells. After 293T cell infection at 0.1 MOI with Sabin2-eGFP, SIV_mac239_CA-p6-OPV and HIV_BG505_C1-V2-OPV, supernatants were harvested at time points indicated on the X axis and the corresponding titer, obtained by TCID_50_ evaluation, is reported on the Y axis.

SHIV_BG505_-OPV is composed of two recombinant OPVs. To obtain plasmid pSIV_mac239_CA-p6-OPV, nucleotides 1125 (bp 406-1530) of the SIV_mac239_ gag sequence (Accession # M33262) (59) are cloned into the XbaI and SalI sites of plasmid pSabin2-eGFP [a gift of Dr. R. Andino, UCSF (60)], between two poliovirus protease cleavage sites, replacing the *gfp* gene. The 375 amino acid (a. a.) SIV Gag polyprotein, covering, CA, NC and p6 proteins, is expressed intracellularly after virus infection and cleavage of the OPV polyprotein. Plasmid pHIV_BG505_C1-V2-OPV carries a portion of the HIV_BG505_ Env sequence, replacing the *gfp* gene in pSabin2-eGFP OPV. pSabin2-eGFP was modified by removing the *gfp* sequences, and by adding a 21 base pair sequence covering the Thosea asigna virus 2A (T2A) polycistronic NPG/P cleavage site (61) right after the 2A polio protease cleavage site and the G-linker to achieve, after cleavage, the addition of only a proline at the N terminus of a signal peptide, provided by the 23 a.a. of the tissue plasminogen activator (tpA) signal peptide. This signal peptide carries the 22P/A mutation, reported to significantly increase the secretory expression of trimeric proteins (**Figure 1A**) (62). The HIV_BG505_ C1-V2 Env fragment was obtained by PCR amplification of the C1-V2 region from the Expression Vector BG505.W6M.ENV.C2 (NIH-AIDS Reagent Program cat no. 11518) and includes the sequence for amino acids 30-209 of HIV_BG505_ Env (sequence accession ABA61516, DQ208458.1) (57), cloned 3’ of the signal peptide sequence. At the 3’ end of the Env sequence, a sequence for a His tag was inserted to detect and purify the secreted protein fragment. The HIV Env C1-V2 fragment, expressed in this virus as part of the OPV polyprotein, is cleaved from the OPV polyprotein and independently secreted from the infected cell. Virus production in HeLa cells and titration of virus stocks were carried out according to Burril, C. P et al. (63)

The following bNAb, utilized to characterize the new recombinants expressing HIV Env fragments, were obtained through the NIH AIDS Reagent Program, Division of AIDS, NIAID, NIH: Anti-HIV-1 gp120 Monoclonal (PG9) from the International AIDS Vaccine Initiative (IAVI, cat# 12149) and Anti-Human Immunodeficiency Virus (HIV)-1 gp120 Monoclonal Antibody (PG16), cat. # ARP-12150, also contributed by IAVI (64).

### Vaccine Formulation, Vaccination Groups and SHIV_BG505_ Rectal Challenge

Sixteen female Rhesus macaques were housed at Biomere Biomedical Research Models, Worcester, MA, according to an approved protocol under the guideline established by the Animal Welfare Act and the National Institute of Health Guide for the Care and Use of Laboratory Animals. They were divided into 3 groups, each of 8 animals. Group 1 and Group 2 animals received a total of three DNA doses of DNA plasmid pSHIV_BG505_Vacc3 on day 1, week 8, 16 that consisted of 1 mg of pSHIV_BG505_Vacc3 DNA, formulated in cationic liposomes as described (56). In addition, on week 33, 41 and 49 Group 1 received rMVA-SHIV-BG505 (10^8^ pfu) and Group 2 received 5x10^7^ pfu SHIV_BG505_-OPV. The boosting schedule was delayed 9 weeks from the original plan (week 24, 32, 40) because of intervening implementation of OPV containment requirements in the facility that housed the animals, requested by the CDC to comply with the WHO program for poliovirus eradication. These vaccines were formulated in PBS in a final volume of 1 ml. The DNA vaccine was administered to the animals in the oral cavity, applied to the mucosa between the gum and the cheek while sedated. OPV is usually given to humans orally and infects both the oral cavity and the gastrointestinal tract after swallowing. This is harder to accomplish in awake animals without any waste of the administered vaccine or prolonged animal training. To be able to compare the administration of the full dose selected for both vaccines and, at the same time, achieve the distribution obtained in humans with OPV, we opted to administer the vaccines partially in the mouth (1/5 of the dose) and the remaining 4/5 in the stomach by gavage for SHIV_BG505_-OPV and in the duodenum using an endoscope for rMVA-SHIV-BG505 [a approached previously tested for SIV-MVA in (56)]. Two animals in Group 2 were euthanized due to rectal prolapse that occurred on weeks 44 and 45 after rectal biopsies obtained on weeks 43, leaving 6 animals in this group. Eight weeks after the last vaccination, Group 1 and Group 2 animals and Group 3 (naïve controls) were inoculated with a 1:75 dilution in PBS of SHIV_BG505_ N332 S375Y DCT stock [a gift from Dr. George Shaw, U. Pennsylvania (30, 65)]. The virus amount corresponded to 1.4 x10^7^ virions or 2 ng p27 and was grown in RM CD3-activated PBMC depleted of CD8 T cells [a complete characterization of the challenge stock with respect to virion content and virion infectivity of the pathogenic virus is provided in S1B in (45)]. SHIV_mBG505_ N332 S375Y DCT was administered non-traumatically with needleless tuberculin syringes as cell-free virus in the rectum (66). Challenge was repeated weekly six times and RT-PCR tests were carried out to detect positivity for virus in plasma.

### Collection of Specimens

Blood and secretions were collected 2 and 4 weeks post-vaccination and were followed by monthly collection. Premoistened Weck-Cel sponges were used to collect rectal and vaginal secretions as described (67). Rectal, vaginal and lymph node tissues were biopsied on the day of first vaccination and 2 weeks after each vaccination. Plasma and PBMC were isolated from EDTA anti-coagulated whole blood using established protocols (68). Isolation of mononuclear cells (MNC) from colon-rectal mucosa was carried out according to previously published procedures (69). Briefly, after Telazol anesthesia, seven to eight biopsies/animal/time point were obtained from the rectum and cervico-vaginal tissue using sterile forceps and a small pinch biopsy device (Olympus endoscopic biopsy forceps). MNC from tissues were obtained by mechanical dissociation using GentleMACS dissociator (Miltenyi Biotech, Paris, France). Suspensions were passed through a 70 mm pore size cell-strainer and washed with 10% RPMI (70).

### Measurement of Antibodies in Plasma and Secretions

Concentrations of antibody to SIVmac239 p27 (Immune Technology, New York, NY) and murine leukemia virus gp70 scaffolded HIV-1 BG505 gp120 V1V2 (gp70-V1V2; from Dr. Abraham Pinter, Rutgers, NJ) were measured using a customized binding antibody multiplex assay (BAMA) as described (71, 72). Briefly, BioPlex Pro magnetic carboxylated beads (Bio-Rad, Hercules, CA) were labeled with p27 or gp70-V1V2 and mixed overnight with serial dilutions of sample and standard. The standard was pooled serum from SHIV-infected macaques, which had been calibrated as described (73). Beads were consecutively washed and treated with biotinylated goat anti-monkey IgA (Rockland Immunochemicals, Pottstown, PA) or anti-human IgG (SouthernBiotech, Birmingham, AL) and neutralite-phycoerythrin (SouthernBiotech). A Bio-Rad Bioplex 200 was used to measure fluorescence intensity and construct standard curves for interpolation of antibody concentrations. Total IgA and IgG concentrations in secretions were measured by ELISA as described (74) using rhesus dimeric IgA (NHP Reagent Resource) and rhesus IgG (Antibodies Inc, Davis, CA) as standards. The concentration of anti-p27 or anti-gp70-V1V2 IgA or IgG in each secretion was divided by the concentration of total IgA or IgG to obtain the specific activity (ng antibody per µg immunoglobulin). The secretion was considered antibody-positive if it had a specific activity that was greater than the mean specific activity + 3 SD in secretions of naïve animals. If a preimmunization secretion had no detectable antibody, it was assigned the mean specific activity value of naive macaques. Concentrations of specific IgG in plasma were considered significant if they were 3.4-fold greater than that measured in the animal’s pre-immune plasma. OPV neutralization titer in RM plasma was determined according to procedures in (75).

### Immunophenotyping and Intracellular Cytokine Staining (ICS)

10^5^ MNC or 10^6^ PBMCs were incubated for 14 hours with medium (unstimulated), 1μg/ml pools of 15-mer SIV Gag or HIV Env peptides (Peptide Pool, SIVmac239 Gag Protein, ARP-12364, contributed by DAIDS/NIAID; Peptide Pool, HIV Type 1 Subtype C (Consensus) Env Region, ARP-12634, contributed by DAIDS/NIAID). Cells incubated with 10 ng/ml PMA (4-α-phorbol 12-myristate 13-acetate; Sigma) and 1μg/ml ionomycin (Sigma) or without any stimulation provided respectively positive and negative controls. Cultures contained BrefeldinA (BD GolgiPlug Cat. # 555029; BD Biosciences) and 1mg/ml of anti-CD49d and anti-CD28. Cells were washed, stained for surface markers in the dark, followed by fixation and permeabilization. After the permeabilization, cells were intracellularly stained for cytokine expression with anti-cytokines antibodies for 1 hour in the dark according to previously described procedures (74). The following antibodies were used in this study and, unless otherwise stated, were purchased from BD Bioscience, San Jose, CA: anti-CD3-pacific Blue (clone SP34-2), anti-CD4-V500 (clone L200, anti-CD8-APC-Cy7 (clone RPA-T8), anti-TNF-α-PE (clone MAb11), anti-IFNγ-Alexa Fluor-700 (clone B27), anti-IL-2-APC (Clone MQ1-17H12), anti-CD95-FITC (DX2) and anti-CD28-PE-Cy5 (clone CD28.2), anti-IL-17-PerCP-Cy5.5 (clone eBio64DEC17, eBioscence, San Diego, CA), anti-Foxp3-FITC (clone 206D, BioLegend, San Diego, CA). For MNC, 200 μl of a 1:100 dilution of the viability dye stock (VIVID, LIVE/DEAD kit, Invitrogen) were added to the antibody cocktail to exclude dead cell background. The acquisition of cells was done on LSRII flow cytometer using FACSDIVA software. The data were analyzed using FlowJO version 10.7.1 software (TreeStar, Ashland, OR). Data for peptide-stimulated populations are reported as percentage, determined after subtracting the percentage of positive cells detected in unstimulated cells for each sample. Evaluation of single, double or triple positive cells, was carried out using FlowJo Boolean gate.

### Viral Load Quantitation

Plasma SIV RNA levels were measured by real-time RT-PCR assay as described (76, 77). The Lifson assay has a threshold sensitivity of 30 copy equivalents per milliliter. Inter-assay variation is <25% (coefficient of variation). Mean viral loads were calculated by transforming the number to its logarithmic value and averaging the logarithmic values of all the animals of the group at one specific time point.

### Euthanasia

Animals were euthanized because of closure of the study or earlier if they developed signs and symptoms consistent with the definition of AIDS. AIDS was defined as being SHIV+ (detectable viremia) and experiencing one of the following criteria: 1- weight loss >15% in 2 weeks or >30% in 2 months; 2- documented opportunistic infection; 3- persistent anorexia >3 days without explicable cause; 4- severe, intractable diarrhea, 5- progressive neurologic signs, 6- significant cardiac and/or pulmonary signs, 7- loss of CD4+ T cells below 200 or 10%.

### Statistical Analysis

Calculations and statistical analyses were performed using the GraphPad Prism version 8 software. Between groups comparisons were carried out by two-tailed, *t* test or Mann-Whitney U test if the value distribution was non- parametric, and among groups one-way ANOVA was used. Results of statistical analyses were considered significant if they produced *p* values ≤ 0.05. Display of multi-component distributions was performed with SPICE v5.2 (freely available from http://exon.niaid.nih.gov/spice/) (78).

## Results

### Engineering of Recombinant OPVs

In previous preclinical trials, we achieved significant systemic and mucosal T-cell responses after rectal, nasal, vaginal, oral and intestinal immunizations with SIV or SHIV DNA combined with SIV- or SHIV-MVA and observed some level of protection, particularly in terms of delayed CD4+ T-cell loss and preservation of the Th17/Treg ratio, but also as significantly higher number of vaginal challenges required to achieve infection (56, 73, 74, 79, 80). However humoral responses were not satisfactory. As poliovirus is capable of stimulating long-lasting humoral responses in humans, and Cynomolgus macaque nasal immunization with 6 doses of a collection of 20 recombinant polioviruses expressing short SIV peptides and covering the SIV *gag, pol, env*, and *nef* genes had provided significant antibody responses, though not NAbs (55), we set out to test recombinant OPVs in combination with a DNA platform to maximize both T and B cell responses in a Rhesus macaque model, recently shown to be infectable orally by poliovirus (81). A previous trial where we explored a SIV immunization with 3 doses of SIV DNA plus SIV-OPV in Cynomolgus monkeys provided significant T-cell responses but very limited anti-SIV antibodies (82). We hypothesized that, among other factors, the intracellular expression of the recombinant fragment expressed by OPV, which does not become part of the virion, may be a limiting factor in the development of a significant antibody response and opted to modify the vector to achieve secretion of the recombinant Env fragment from the poliovirus-infected cells by engineering an OPV vector that could secret recombinant antigens.

The structure of the SIV Gag and the HIV env OPV expression vectors used in the vaccination are reported in **Figure 1A** and in Materials and Methods. SIV_mac239_ CA-p6 sequences inserted in the OPV vector achieve intracellular expression of these Gag proteins. This mode of expression was selected because it is conducive to cell-mediated responses and anti-Gag T-cell responses have been shown to be important for viremia control after infection. Towards the goal of secreting recombinant OPVs, the tPA signal peptide sequence was inserted 5’of the HIV Env sequences and a sequence for the T2A cleavage site was positioned 5’ of the tPA element to obtain cleavage compatible with secretion (61) (**Figure 1A**). Efficient replication and virus stability of HIV**Env**recombinant OPVs constructed in this vector did not occur for a few attempted constructs, possibly due to misfolding of the recombinant OPV precursor, preventing the appropriate cleavage of the polyprotein and therefore virus assembly. Of two replication-competent, stable OPVs expressing the V1V2 HIV region, we selected the construct HIV_BG505_C1-V2-OPV, expressing 179 amino acids of HIV Env_BG505_ and covering the Conserved region 1 (C1) and the V1V2 domain, as antibodies against this region have been shown to be associated with protection in clinical trials (1, 3, 4, 6, 32, 33). SIV_mac239_CA-p6-OPV and HIV_BG505_C1-V2-OPV replicated efficiently over 12 cell passages, as indicated by the detection of the appropriate size fragment by RT-PCR in 293T RNA (**Figure 1B**). Expression of the SIV_mac239_ CA-p6 was detected by Western blot in infected cell lysates and by flow cytometric analysis (**Figure 1C**, left panels). The recombinant HIV_BG505_C1-V2 fragment was detected by flow cytometry in infected cells and could be immune-precipitated using the bNAb PG16 but not PG9 (64) after its purification from tissue culture supernatant (**Figure 1C**, right panels). These data support the conclusion that secretion of the recombinant fragment could be achieved from cells infected with the modified OPV vector and that the secreted product retained the conformation necessary for the recognition by a bNAb, supporting the *in vivo* occurrence of the same conformation as well. Lastly, we evaluated whether the two OPV recombinants retained *in vitro* replication kinetics similar to those of the reference virus Sabin2-eGFP by evaluating the growth curve of these viruses in 293T cells. Values for infection at MOI of 0.1 are reported in **Figure 1D**. No significant differences were observed for SIV_mac239_CA-p6-OPV and HIV_BG505_C1-V2-OPV that were selected for the recombinant OPV boosting and, mixed at a 1:1 ratio, are collectively defined as SHIV_BG505_-OPV.

### Oral Vaccination Stimulates Systemic and Mucosal Anti SHIV Responses

**Figure 2:**
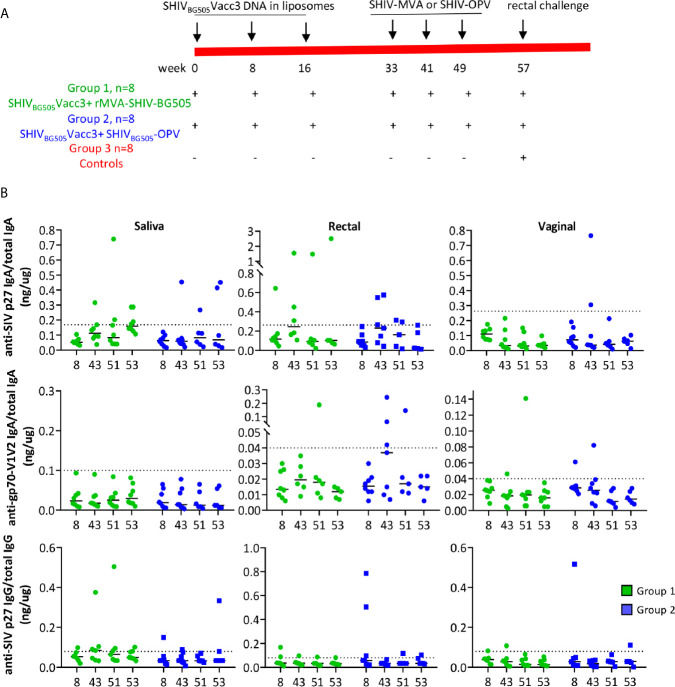
**(A)** Vaccination scheme and animal groups. **(B)** Levels of anti-SIV p27 IgA (top 3 panels), anti-gp70-V1V2 IgA (middle panels) and anti-SIV p27 IgG in saliva (left column), rectal (middle column), and vaginal secretions (right column) were measured by BAMA and normalized relative to concentrations of total IgA or IgG, determined by ELISA. The specific activity (ng IgA or IgG antibody/µg total IgA or IgG, respectively) for individual animals at four time points during vaccination is reported. The dashed line denotes the cut-off for significance and was calculated as the mean specific activity + 3 SD for pre-immunization samples. The specific activity had to exceed the dotted line and be 3 times higher than the animal’s pre-immune sample to be considered significant. A few samples with insufficient total IgA or IgG were not tested for SHIV-specific antibodies.

We evaluated stimulation of systemic and mucosal cell-mediated immunity and humoral immunity in Rhesus macaques that, contrary to previous reports, were shown to be infectable by the poliovirus (81). The two vaccine regimes included as primer the DNA plasmid pSHIV_BG505_Vacc3, producing non-infectious viral particles, formulated in liposomes, and administered in the oral cavity, boosted in one group by three doses of rMVA-SHIV-BG505, expressing SIV_mac239_ Gag/Pol and HIV_BG505_ Env, and in the other group by SHIV_BG505_-OPV, both administered in the oral cavity and in the gastrointestinal tract according to the vaccination schedule illustrated in **Figure 2A**. The rationale for the choice of vaccine components is the following: anti-HIV and SIV T-cell mediated immunity, particularly anti-Gag, which is achieved with DNA immunization, has been shown to provide control of viremia and to delay disease progression, which are desirable features of an HIV vaccine (73, 79, 80, 83). The boosting with two recombinant OPVs, one expressing SIV Gag and one expressing HIV Env, was based on the rationale of expanding responses primed by the DNA and, in the case of the Env immunogen, covering the V1V2 domain to stimulate antibody responses against this region, whose binding antibodies have been shown to correlate with protection from infection (5, 33). As the conformation of the C1-V2 fragment after cell expression and secretion is recognized by the bNAb PG16, this antigen appears to retain a V1V2 conformation potentially able to induce bNAbs. As the C1 region has been shown to be targeted by ADCC mediating antibodies (5, 84, 85) having it included provided the possibility of generating ADCC antibodies against this domain. During the time course of the vaccination, systemic and mucosal antibody responses were evaluated in plasma and secretions (**Figure 2B**). Stimulation of antibody responses was disappointing in both groups with anti-SHIV IgG in blood being negative and anti-SIV p27 IgG in secretion being positive in only a few animals (**Figure 2B**, bottom panels). Positivity for anti-SIV p27 or gp70-V1V2BG505 IgA in saliva, rectal, vaginal secretions was restricted to one or two animals in each of the two groups in some of the tested time points. We may have missed antibodies recognizing SIV pol or SIV NC and p6, as these were not included as test antigens in the multiplex antibody assay. Regardless, the SHIV-OPV immunization did not achieve the significant antibody responses we had expected. Although disappointing, the fact that some animals did respond suggested that the vaccines can stimulate antibody responses and that a higher dose might be necessary to achieve more uniform, significant results. Interestingly, we could not detect any neutralizing OPV titer in the plasma of the animals that received the recombinant OPV, when tested in a neutralization assay against the parent virus Sabin2-eGFP. These results point more directly to this immunization being low in dose, as plasma anti-OPV antibody titers have been reported in Rhesus Macaques only when OPV viremia is achieved, this required doses equal or higher than 10^8^ and did not occur in animals infected with a dose of 10^7^ TCID_50_ OPV (81). It appeared that detection of neutralizing antibodies correlated with the resolution of viremia (81). In humans, prolonged infection and shedding, a parameter we did not measure, correlated with high titers of neutralizing antibodies (54) and prolonged infection may be necessary to achieve the transient viremia critical to induction of neutralizing antibodies. Our dose of 5x10^7^ TCID_50_ OPV, half of each for each recombinant, was selected to avoid the possibility of poliomyelitis that was observed more frequently in macaques receiving higher doses (81) and would have required euthanasia.

**Figure 3:**
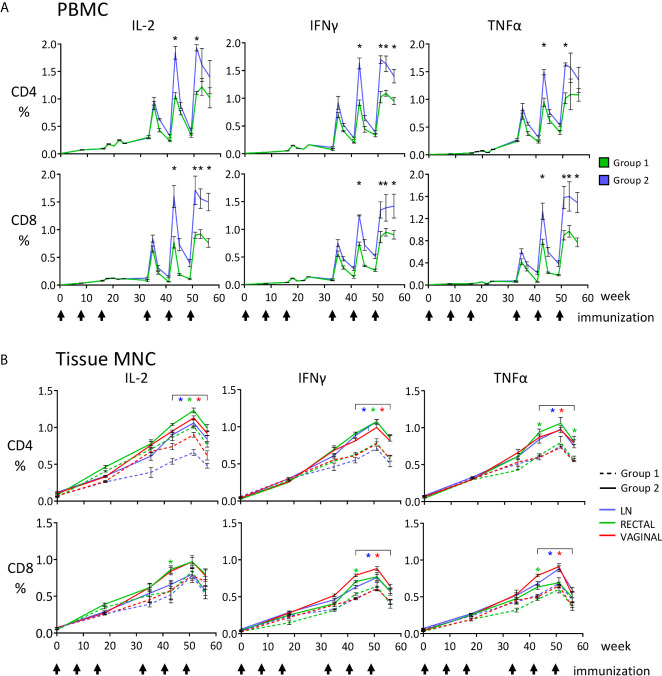
Quantitative analysis of the anti-SHIV cell-mediated responses measured during the immunization phase, reported as percentages of CD4+ and CD8+ T cells producing IFNγ, TNF-α and IL-2, detected by ICS and flow cytometric analysis upon stimulation with SIV Gag or HIV Env peptide pools. **(A)** PBMC, asterisks indicate that T-cell percentages (Gag + Env) at the indicated time points are significantly higher for Group 2 (SHIV_BG505_-DNA+SHIV _BG505_-OPV, blue) compared to Group 1 (SHIV_BG505_-DNA+ rMVA-SHIV-BG505, green); **(B)** LN, rectal and vaginal MNC; the graphs show the total SHIV-specific T-cell responses (Gag + Env) for the two vaccinated groups, Group 1 (dashed line) and Group 2 (solid line). Color refer to the source of samples examined (LN, vaginal or rectal). The color of the asterisks used in panel **(B)** to report statistical significance matches the color used for the samples in question. Asterisks under brackets indicate that values from week 43 to week 56 are all significantly higher for the specific tissue when Group 2 is compared to Group 1 (p value range: 0.0001-0.039). Between groups comparisons reported in panels **(A, B)** were carried out by two-tailed, *t* test or Mann-Whitney U test when the value distribution was non-parametric.

**Figure 4:**
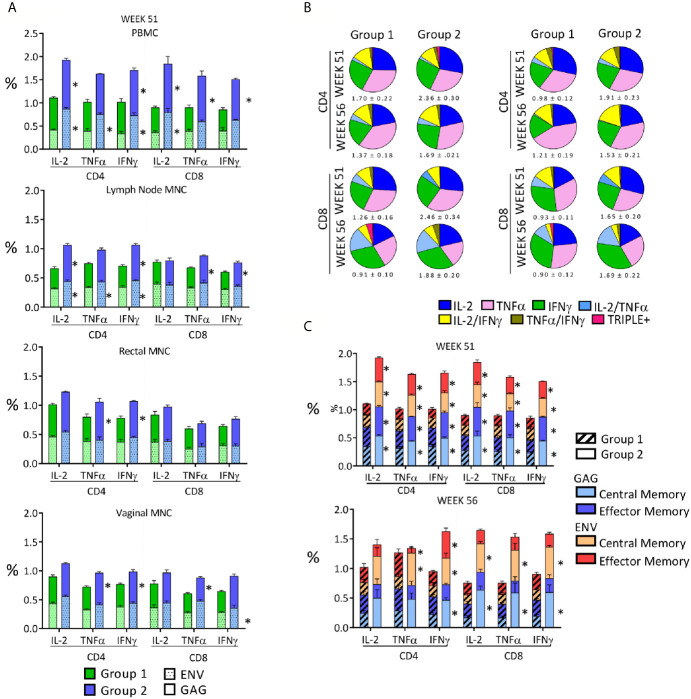
Qualitative analysis of the anti-SHIV cell-mediated responses stimulated by the vaccination. **(A)** Anti**-**SIV Gag and Env percentages observed 2 weeks after the last immunization in the two vaccinated groups in PBMC, LN, rectal and vaginal MNC. Asterisks indicate significant difference between Group 1 and Group 2 and are placed adjacent to the relevant column segment. **(B)** Pie graphs representing the diversity of anti-SHIV CD4+ and CD8+ T-cell responses as relative fractions of the total percentages of positive cells, expressing a IFNγ, TNF-α and IL-2 alone or a combination of them within anti-SIV gag (left panel) or anti-HIV env (right panel) responses. Functional analysis is in biopsies 2 weeks after the last immunization (week 51) and two weeks before challenge (week 56). The total mean percentage and SE of the antigen-specific responses for each analyzed variable is shown below each pie. **(C)** Central memory (C_M_) and effector memory (E_M_) fractions in SHIV-specific cell-mediated T-cell responses present in PBMC on week 51 (2 weeks after last immunization) and week 56 (naïve cells: CD28+CD95-; memory T cells: CD28+CD95+; and effector T cells: CD28-CD95+). The average percentages of anti-Gag and anti-Env T cells in each group are stacked in the column and shaded differently. Asterisks adjacent to column segments indicate a significant difference between Groups 1 and 2 for that segment. Between groups comparisons reported in panels **(A, C)** were carried out by two-tailed, *t* test or Mann-Whitney U test if the value distribution was non- parametric (p value range: 0.0001-0.045).

During a time-course analysis, we evaluated anti-SHIV CD4+ and CD8+ T-cell responses in PBMC, lymph node (LN), rectal, and vaginal MNC by evaluating anti-SIV Gag and anti-HIV Env responses using cytokine intracellular staining after peptide stimulation and flow cytometric analysis. Significant cell-mediated responses were detected in all analyzed compartments after immunization with both regimes (**Figure 3**). Each boosting immunization increased the previously observed levels of cell-mediated responses that were at very low levels in blood after the third DNA dose (**Figure 3A**), but more significant in rectal and vaginal mucosal MNC (**Figure 3B**). This result was expected, as responses are usually higher at the site of immunization and of lower magnitude at other sites due to the compartmentalization of the immune system, particularly between mucosal and systemic compartments, and therefore one is unlikely to achieve in blood what is observed in the gastrointestinal tract after oral immunization (44–50). The SHIV_BG505_-OPV boosting immunization stimulated levels of responses that were significantly higher than those observed with the rMVA-SHIVBG505 in all analyzed compartments for most CD4+ and CD8+ T-cell responses evaluated on week 43, 51 and 56 (**Figure 3**, p value range: 0.0001-0.039). The relative contribution of anti-SIV Gag and anti-HIV Env responses, reported as their sum in **Figure 3**, is shown in **Figure 4A** for week 51 PBMC, two weeks after the last immunization. Interestingly, although only shorter SIV Gag and HIV Env protein fragments were included in the recombinant OPV construct compared to the rMVA-SHIV-BG505 construct that includes the entire Gag/Pol and Env gp120, significantly higher anti-Gag and anti-Env responses were observed with the OPV-based vector at the end of the immunization (**Figure 4A**, p value range: 0.009-0.045, depending on cytokine and sample). Boolean analysis of the expression of TNF-α+, IFNγ+ and IL-2+, in antigen specific cells revealed predominantly monofunctional responses for the 3 cytokines tested, but multifunctional responses ranging between 20 and 27% of the total were also present in all tissues and no major differences in mono- or polyfunctionality distribution were observed between the two groups (**Figure 4B**).

We compared the central memory (C_M_) and effector memory (E_M_) subset fractions of the cell-mediated response in PMBC and tissue MNC 2 weeks after the last immunization (week 51) and 7 weeks after the last immunization (week 56). Roughly equal distribution of C_M_ and E_M_ responses was present in anti-SIV Gag and anti-HIV Env responses on week 51 in both groups and the percentages shifted in favor of the C_M_ responses by week 56 in both groups (**Figure 4C**), supporting the expected contraction of the E_M_ component and capability of the vaccination regimens to maintain antigen-specific C_M_. Many percentages of C_M_ and E_M_ were significantly higher in Group 2 when compared to those of Group 1, especially at week 51 (P value range: 0.0001-0.043, **Figure 4C**). Interestingly, vaginal T-cell responses were comparable to those detected in the rectum, supporting this platform of immunization as a way to achieve responses at two sites that are highly significant in HIV transmission.

When all above data are considered together, we concluded that the two vaccine modalities given orally were effective in stimulating cell-mediated T-cell responses at multiple sites, including those of HIV exposure, but significantly less effective at stimulating humoral responses. These data indicate that the oral route can be used as route of vaccination to stimulate broad T-cell responses at mucosal sites relevant to HIV entry, and also systemic sites as blood and lymph nodes. However, the recombinant OPV and MVA at the dose and route used here were insufficient to stimulate consistent and high titer antibody responses to the recombinant antigens and neutralizing antibodies to OPV. As antibodies to SIV fragments shorter than those used here, expressed by recombinant OPVs, were induced after six nasal doses in the study by Crotty et al. (55), the reason for our results remains unclear but it may have been due to administration of too low of an oral dose.

### Resistance to Challenge and Disease Progression

**Figure 5:**
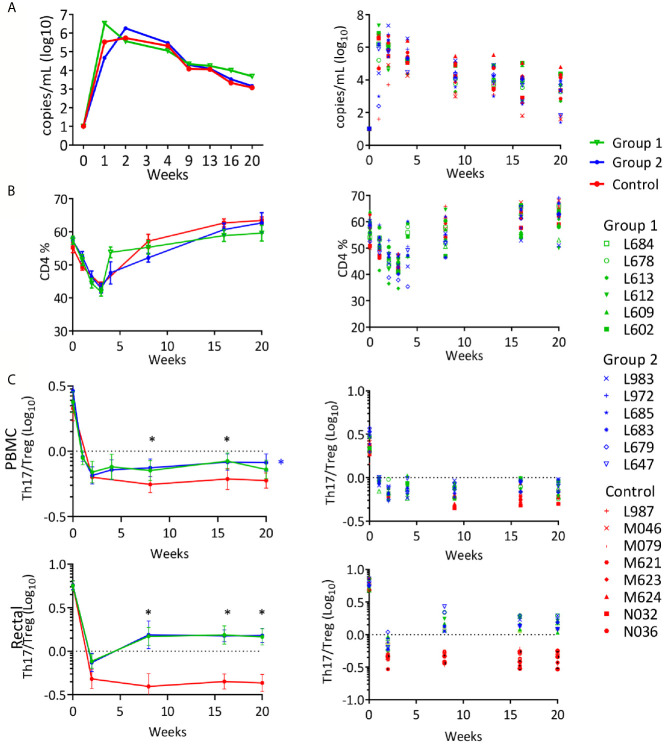
Rectal SHIV_BG505_ challenge outcome: **(A)** Viral loads reported as geometric group means (Log_10_) in the left panel and for each individual animal in the right panel. **(B)** PBMC CD4+ T-cell levels reported for each group as average percentages (left panel) or as percentages for each animal (right panel) during the course of the infection. **(C)** Th17/Treg ratio in PBMC (top) and rectal MNC (bottom) CD4+ T cells, reported as group average ± SE (left panel) or as individual values for each animal (right panel) during the course of the infection. Statistical significance among groups was tested with 1-way ANOVA with Sidak’s multiple comparisons test (*p value range: 0.0001-.021). Black asterisks indicate time points when both Group 1 and Group 2 values were significantly greater than those in the control group. The blue asterisk in the PBMC panel indicates significance for Group 2 value *vs.* control group.

To evaluate whether the vaccination could provide protection from infection or disease progression, on week 57, 8 weeks after the last immunization, all vaccinated animals and eight naïve female controls received a rectal challenge with SHIV_BG505_ (37). As previously observed for control animals rectally inoculated with the same dose of SHIV_BG505_ (37), all controls became infected after a single dose. Among vaccinated animals, 11 animals became infected after one dose, one animal became infected after 2 doses and two animals, both in the SHIV DNA/MVA vaccine group, resisted six challenges, after which no more challenges were administered. No significant differences were observed between groups when post-infection viral loads were evaluated in the infected animals (**Figure 5A**). The control group obtained a better control of SHIV viremia than what we have observed with SIV and recovery to normal level of CD4+ T cells occurred in this group as in vaccinated animals (**Figure 5B**). However, when the ratio of Th17/Treg was investigated at multiple time points after infection, both vaccinated groups recovered from the initial decrease to a better ratio than controls during the chronic phase of the infection and this was true in PBMC and in rectal MNC (**Figure 5C**). The differences between Group 1 or Group 2 *vs.* Controls were statistically significant (p value range: 0.0001-0.021) on week 8, 16 and 20, with the only exception being week 20, PBMC samples, where differences were significant for Group 2 compared to controls but not for Group 1 *vs.* controls. These results may reflect a better recovery and subsequent preservation of the Th17 cells that are lost in the intestine during HIV and SIV infection, supporting protection by the vaccination of the heavily targeted intestinal immune compartment. This parameter has been shown to be the most accurate predictor of disease progression (86–89) and it is possible that delayed occurrence of disease could have happened in the vaccinated groups if kept for a longer period of time under observation.

## Discussion

Significant protection from SIV infection but not from disease progression was previously observed in Rhesus macaques after intestinal immunization and the reverse was true for oral cavity immunization with a vaccine made of non-infectious proviral SIV DNA plus SIV-MVA (79). We hypothesized that an immunization at both sites (oral cavity and intestine) may achieve both outcomes and that a recombinant OPV vaccine could be a good vector to achieve this goal, considering its capacity to replicate in the oral cavity and in the entire gastrointestinal tract. In addition, OPV should provide significant stimulation of humoral responses, both mucosally and systemically. We compared this platform to a previously tested boosting of DNA immunization with recombinant MVA (79), this time administering it both orally and intestinally. Significant responses were observed for stimulation of cell-mediated immunity at both rectal and vaginal sites, where most HIV transmissions occur. Antigen-specific pre-existing immunity permitted a better recovery of the Th17/Treg ratio, one of the most significant predictors of disease progression after infection and a parameter highly dependent on preservation of intestinal Th17 cells. Although measuring tissue-resident memory T cells was beyond the scope of this trial, it is very likely that an approach that utilizes a gastrointestinal route of vaccination will establish a higher percentage of antigen-specific tissue-resident memory T cells (Trm) in the intestinal mucosa. These cells do not recirculate, can immediately be activated when pathogen breaches the mucosal barrier without the need for antigen presentation in lymph nodes, and they are the largest contributor to the expansion of the response upon restimulation (90–93). Trm can therefore more promptly control local infection. In a smallpox skin reinfection model, circulating memory T cells cleared the infection after 26 days while only 6 days were required for clearance by Trm cells (94). The availability of antigen-specific Trm cells in the rectum could be particularly important for preventing rectal transmission of HIV.

Results of humoral responses with recombinant SHIV_BG505_-OPV were disappointing. A number of reasons could justify the results, the most likely being a low dose and/or number of immunizations, as sporadic antibodies to SHIV antigens could be detected in some animals, supporting the capability of the immunogen to stimulate antibody responses. Antibody production is known to require higher amounts of antigen than those required for stimulation of cell-mediated immunity (95). The capability of recombinant OPV to induce antibodies is supported by the study by Crotty et al. (55), where antibodies to recombinant OPV expressing fragments shorter than those used here were observed after six doses administered *via* the nasal route, a route that is known to be better than other mucosal routes at inducing systemic immunity. This result support the capability of obtaining antibodies to a recombinant peptide expressed in the context of the OPV vector. However, the 179 amino acid Env fragment of the ~480 amino acid gp120 Env protein, inserted in the OPV recombinant used here, could be poorly suited to generate antibodies when expressed in the context of OPV infection. Additional support for an insufficient total dose of recombinant OPV vaccination comes from the fact that we could not detect neutralizing antibodies against OPV that usually occur after OPV vaccination nor against the 375 amino acid Gag fragment that covers 75 percent of the Gag polyprotein. Dose-escalation experiments with immunizations of the recombinant OPVs used here administered *via* either the gastrointestinal route or the nasal routes might address this issue and determine if higher doses or a nasal administration of this vaccine provide the ideal platform for stimulation of significant humoral responses in addition to cell-mediated responses, or if these recombinants are simply not suited for the induction of antibodies.

Ideally, a dose-escalation study would be necessary to figure out the most appropriate dose to be administered. We employed the dose reported with the minimal side effects in Rhesus macaques to avoid having to euthanize animals after vaccination, if paralysis occurred (81). The dose was half for each recombinant OPV and it is possible that each needs to be used at a higher dose.

The combined use of the SHIV_BG505_-DNA and rMVA-SHIV-BG505 did not provide the protection from infection that we had observed in an oral SIV-Rhesus model of intestinal vaccination with the same DNA/MVA modality, where 50% of the animals resisted 32 vaginal challenges in a trial where the median number of challenges for control was 8 (64), although in this trial, 2 of 8 Group 1 animals (25%) did not become infected after exposure to 6 challenges when instead one challenge was sufficient to infect all control animals. In that investigation, challenge was vaginal and not rectal and the dose of SIV_mac251_ used for repeated low dose challenge was most likely lower, considering the number of challenges that were required to infect the controls. We also noticed that in this trial the reduction of SHIV_BG505_ viremia from peak to the chronic levels was more significant in controls than in SIV_mac251_ infected macaques. This is reflected in the recovery of CD4+ T cell percentages to normal values in all controls as well as in vaccinated animals, making it difficult to reveal a protective effect of the vaccine with this parameter. In the study by Jones et al. (96), antibody development and partial protection against rectal challenge were observed after oral vaccination with HIV-MVA combined with recombinant trimeric gp120. In this case, HIV-MVA was administered *via* a needle-free injector and this tool, also used to deliver oral anesthesia, allows for systemic exposure of the antigens, favoring a different stimulation of the immune system.

Despite inefficient stimulation of antibodies, detection of significant antigen-specific T-cell responses after Gag or Env stimulation of macaque MNC indicates that both recombinant OPVs expressed the Gag and Env fragments *in vivo* in amounts sufficient to stimulate cellular immunity. The SHIV_BG505_-OPV vaccine stimulated cell-mediated responses both at mucosal and systemic sites, a parameter known to be achievable at lower doses of immunogen, yet sufficient to confirm the immunogenicity of the vaccine. Many parameters of cell mediated immunity were significantly higher in the SHIV_BG505_-OPV vaccinated animals than in those given rMVA-SHIV-BG505, particularly when it comes to vaginal immune responses that could be useful when viral exposure is vaginal. Importantly, SHIV_BG505_-OPV, like rMVA-SHIV-BG505, permitted a reduced loss and better recovery of Th17+/CD4+ T cells, as indicated by a significantly better CD4+ Th17/Treg ratio compared to controls observed in the chronic phase of the infection, supporting functional efficacy of the induced cell-mediated immunity. This outcome most likely reflects a prompter, earlier control of viral replication in the gastrointestinal mucosa where the bulk of Th17 loss occurs even when antiretroviral therapy is administered and maintained (97, 98). It is likely that the oral route of vaccination favors this outcome, as it has been shown that mucosal immunity occurs at the highest level at the site of immunization (51). Unfortunately, plans to evaluate viral loads in the GI tract during the infection were not in place and therefore direct data for this parameter are not available.

The simultaneous presence of both mucosal and systemic humoral and cell-mediated immunity could be important in preventing the establishment of a chronic HIV/SIV infection and in controlling viremia and disease progression when chronic infection occurs. Immunologically-mediated containment of local infection during its initial local phase (or eclipse phase, as the virus in not detected in the plasma) might be possible even in the absence of sterilizing immunity, and humoral mucosal immunity could be critical to achieve this goal before a large-scale anamnestic immune response occurs (99). If control of HIV or SIV infection needs to be achieved at the site of exposure, before a chronic systemic infection becomes established, it is important to have persistence of virus-specific antibodies in addition to memory cell-mediated mucosal immunity that requires activation and responds more slowly (18, 100, 101). Secreted antibodies might provide the first line of defense against the virus inoculum and local interstitial antibodies could act as the second line of defense against virus that succeeds at entering the mucosa together with virus-specific cytotoxic T cells (53, 102, 103). Regional immunity could eliminate any residual infectivity. Once chronic viremia is established, the immunity provided by vaccination would be important to contain virus replication and delay disease as clearance of infection appears to be extremely difficult, even when the infection occurs with highly attenuated viruses (104, 105). As a significant anamnestic response takes longer than the limited amount of time that usually occurs between exposure and systemic dissemination, the intervention of multiple arms of the vaccine-induced immunity may be one way to reduce within manageable limits the magnitude of the local infection and prevent chronic systemic infection. Most HIV infections are sexually transmitted and if immune responses are needed in both the genital tract and rectum, mucosal immunization may provide this outcome more likely than systemic immunization (51).

Considering the rationale illustrated above and the immunogenicity data of SHIV-OPV provided here, it seems reasonable that recombinant OPVs receive a further evaluation where a higher amount of delivered vaccine is achieved either with three higher doses or with more than three doses similar in amounts to those used here. The persistence of antibodies provided in humans by OPV, and the maturation of the response achievable with a replicating vector that provides prolonged stimulation for days after inoculation, could provide the ideal vector to deliver the HIV Env immunogen most suited to induce bNAbs once it is identified.

## Data Availability Statement

The raw data supporting the conclusions of this article will be made available by the authors, without undue reservation.

## Ethics Statement

The animal study was reviewed and approved by Institutional Animal Care and Use Committee at Biomere Biomedical Research Models, Worcester, MA.

## Author Contributions

EV: Flow cytometric evaluation of cell-mediated immunity during the immunization and post-challenge immune system assessment, statistical analyses. LW: construction of SIV_mac239_CA-p6-OPV plasmid, characterization of recombinant OPVs and generation of recombinant OPV stocks for vaccination, post-challenge qualitative viral load evaluation. DB: SHIV Env replacement and Western blot analysis of pSHIV_BG505_Vacc3, construction of HIV_BG505_C1-V2-OPV plasmid, flow cytometry data entry review. SG and RA: construction and production of rMVA-SHIV-BG505 vaccine. RW: antibody measurement assays. PK: evaluation of humoral immunity, manuscript revision. AA: conceptualization, supervision of experiments and data analysis, manuscript writing, project management and funding acquisition. All authors contributed to the article and approved the submitted version.

## Funding

This work was supported by NIH grant R01DE26325 to AA and UM1 AI124436 to RA.

## Conflict of Interest

The authors declare that the research was conducted in the absence of any commercial or financial relationships that could be construed as a potential conflict of interest.
